# Supplementary material for: Pretargeted Alpha Therapy of Disseminated Cancer Combining Click Chemistry and Astatine-211
Source: Pharmaceuticals (Basel). 2023 Apr 15;16(4):595. doi: 10.3390/ph16040595 (PMC10145095; doi:10.3390/ph16040595)
Supplement: Supplementary file 1 [file pharmaceuticals-16-00595-s001.zip › pharmaceuticals-2312519-supplementary.pdf]

Supplementary material

Pretargeted Alpha Therapy of Disseminated Cancer combining Click Chemistry and Astatine-211

Chiara Timperanza[1]*, Holger Jensen[2], Tom Bäck[1], Sture Lindegren[1], Emma Aneheim[1,3]

[1] Department of Medical Radiation Sciences, Institute of Clinical Sciences, Sahlgrenska Academy, University of Gothenburg, 413 45 Gothenburg, Sweden;

[2] PET and Cyclotron Unit, KF-3982, Copenhagen university hospital, DK2100 Copenhagen, Denmark;

[3]Department of Oncology, Sahlgrenska University Hospital, Region Västra Götaland, 413 45 Gothenburg, Sweden;

* Correspondence: chiara.timperanza@gu.se

Table S1: Blood activity profiles reported as the percentage of injected activity per gram (%IA/g) over time in healthy female Balb C mice of two sizes of iodinated effector molecule: 21 kDa (average from n = 2; A and B) and 10 kDa (n = 1; C) poly-L-lysine.

	%IA/g		
	21 kDa		10 kDa
**Time (h)**	**A**	**B**	**C**
0,4	3,4	3,6	3,4
0,8	3,0	2,2	2,5
1,9	1,3	1,7	0,8
3,9	0,7	0,5	0,5
20,5	0,1	0,1	0,1

Table S2: Biodistribution of two sizes of iodinated effector molecule, 21 kDa and 10 kDa poly-L-lysine, reported as the percentage of injected activity per gram (%IA/g) over time in four healthy female Balb C mice (two mice for each group). One of the animals injected with the 10 kDa effector molecule received an extravascular injection and was therefore excluded from the study.

	%IA/g after 24 h	
**Organ**	**21 kDa**	**10 kDa**
Blood	0,02 ± 0,01	0,04
Heart	1,18 ± 0,04	0,06
Lungs	1,50 ± 1,02	0,13
Salivary glands	0,06 ± 0,01	0,02
Throat (incl. Thyroid)	0,49 ± 0,09	1,13
Liver	17,35 ± 0,88	5,82
Spleen	4,06 ± 0,25	1,41
Kidneys	0,39 ± 0,04	0,21
Stomach	0,16 ± 0,04	0,02
Small intestine	0,09 ± 0,09	0,03
Large instestine	0,11 ± 0,04	0,08
i.p. fat	0,15 ± 0,10	0,05
Muscle	0,56 ± 0,35	0,02
Femur	1,20 ± 0,20	0,51
